# Iron Deficiency and Bariatric Surgery

**DOI:** 10.3390/nu5051595

**Published:** 2013-05-15

**Authors:** Ignacio Jáuregui-Lobera

**Affiliations:** Department of Molecular Biology and Biochemistry Engineering (Nutrition and Bromatology), Pablo de Olavide University, Seville 41013, Spain; E-Mail: ignacio-ja@telefonica.net; Tel.: +34-954-280-789; Fax: +34-954-278-167

**Keywords:** obesity, bariatric surgery, iron deficiency, iron deficiency anaemia, nutritional assessment, nutritional supplementation

## Abstract

It is estimated that the prevalence of anaemia in patients scheduled for bariatric surgery is higher than in the general population and the prevalence of iron deficiencies (with or without anaemia) may be higher as well. After surgery, iron deficiencies and anaemia may occur in a higher percentage of patients, mainly as a consequence of nutrient deficiencies. In addition, perioperative anaemia has been related with increased postoperative morbidity and mortality and poorer quality of life after bariatric surgery. The treatment of perioperative anaemia and nutrient deficiencies has been shown to improve patients’ outcomes and quality of life. All patients should undergo an appropriate nutritional evaluation*,* including selective micronutrient measurements (e.g., iron), before any bariatric surgical procedure. In comparison with purely restrictive procedures, more extensive perioperative nutritional evaluations are required for malabsorptive procedures due to their nutritional consequences. The aim of this study was to review the current knowledge of nutritional deficits in obese patients and those that commonly appear after bariatric surgery, specifically iron deficiencies and their consequences. As a result, some recommendations for screening and supplementation are presented.

## 1. Introduction

Morbid obesity (MO) has been defined as a chronic and multifactorial disease associated with remarkable physical and psychological complications, which can negatively affect quality of life and shorten life expectancy [1]. The indication of bariatric surgery (BS) as therapeutic procedure for MO requires the application of selection criteria, which deal with the degree of obesity, associated complications and previous failure of conventional therapy [2]. The prevalence of obesity in recent years has shown an alarming increase. The repeated failure of balanced food patterns, physical activity, nutritional education and pharmacotherapy have led to the need to use BS both to maintain long-term weight loss, and for the resolution of associated comorbidities [3].

The surgical procedures related to obesity started in the mid 20th century with the first malabsorptive technique. Since then more than 30 procedures have been developed [4]. Surgical techniques fall into three general categories: gastric restrictive procedures (e.g., gastric band, vertical gastroplasty, *etc.*), malabsorptive procedures (e.g., biliopancreatic derivation, jejuno-ileal bypass, *etc.*) and mixed or combined procedures (e.g., gastric bypass), the latter being the most used procedures [2,5]. Following BS, a relevant improvement in associated comorbidity can be demonstrated, especially in what refers to diabetes, hypertension, dyslipidaemia and apnoea [2]. In 2008, more than 200,000 people had BS in United States with a general prevalence of perioperative complications <1% (surgical procedures for BS with morbidity <10% and mortality <1% are considered as safe procedures) [4,6]. Nevertheless, the postoperative complications are usually quite high taking into account that BS often involves gut manipulation that alters the natural absorption of nutrients, thus causing possible nutritional deficiencies [6]. 

Apart from the consequences of BS, it is worth mentioning that obesity-induced chronic inflammation leads to activation of the immune system, which causes iron-homeostasis alterations. Thus, preoperative anaemia and iron deficiencies are common among obese patients about to undertake BS [7]. In addition to pre- and postoperative complications, it must be noted that BS is a long-lasting inflammatory stimulus in itself, which may cause problems since the perioperative period (e.g., blood loss, induced inflammation, *etc*.). It has been estimated that the prevalence of anaemia in patients scheduled for BS is 10%–15% and the prevalence of iron deficiencies (with or without anaemia) may be higher (up to 30%–40%) [8,9,10,11]. The prevalence of postoperative anaemia and iron deficiencies are thought to be even higher. 

In summary, the presence of nutritional deficiencies in obesity may seem paradoxical (considering the excess caloric intake), but many studies have documented that several micronutrient deficiencies may be higher in prevalence in obese people, particularly in those suffering from extreme obesity (BMI > 40 kg/m^2^). Compounding the problem, BS for MO have also grown in frequency and may exacerbate pre-existing vitamin and mineral deficiencies or produce new ones, depending on dietary intake, adherence to recommended post-operative supplementation and degree of malabsorption associated with the BS procedure [12].

The aim of this study was to review the current knowledge of nutritional deficits in obese patients and those that commonly present after BS, specifically iron deficiencies and their consequences. As a result, some recommendations for screening and supplementation are presented.

## 2. Method

### 2.1. Searching Process

The searching process covered three relevant electronic databases (Medline, EMBASE and Scopus). The general strategy included terms related to BS and iron deficiency. Then the Medical Subjects Headings were used as well as the Boolean operators AND/OR. The shared Mesh terms were (((“Nutritional deficiencies” [Mesh]) OR (“Micronutrients” [Mesh]) OR (“Iron deficiency”[Mesh]) OR (“Iron deficiency anaemia”[Mesh])) AND ((“Bariatric surgery” [Mesh]) OR (“Roux-en-Y” [Mesh]) OR (“Gastric bypass” [Mesh]) OR (“Biliopancreatic bypass” [Mesh]) OR (“Biliopancreatic diversion” [Mesh]) OR (“Banded gastroplasty” [Mesh]))). 

An additional search was carried out on references included in the papers, published reviews and via hand searching. The literature search was limited to articles published between 1995 and 2012.

Studies meeting the following criteria were included in the review, (1) studies focused on BS procedures (Roux-en-Y, Gastric bypass, Biliopancreatic bypass/diversion, Banded techniques) and iron deficiency; (2) controlled trials, randomized controlled trials and comparative studies. Applied exclusion criteria included, (1) descriptive studies or case reports; (2) interventions targeting populations with non-specific iron deficiencies-related problems; (3) participants with special conditions other than obesity (e.g., pregnancy); (4) not available full text/abstract. Reviews and meta-analysis were considered as other source of articles, which fitted the inclusion criteria. An additional search was carried out on references included in the papers and via hand searching.

The initial search yielded 110 references. References were combined in an EndNote 9 library and screened on the basis of title and abstract; those clearly not meeting the review criteria were excluded, as were duplicates. Thereafter, selected references were screened based on full text. Reasons for exclusion were applied and 80 studies were finally included. 

### 2.2. Thematic Analysis

Thematic analysis was used to analyze the papers, the six step framework of Braun and Clarke [13] guiding the process: becoming familiar with the data; creating initial codes; searching for themes; reviewing themes; defining and naming themes and producing the report. Fragments of data that identify a significant feature of such data were acknowledged and grouped together into related themes [13,14]. As a result we obtained the following different topics: follow-up studies *vs.* retrospective studies, follow-up studies after a specific surgical technique, follow-up studies comparing two different surgical techniques, results of a specific technique after the previous failure of another one and nutrition care after BS.

## 3. Results

### 3.1. Retrospective Studies

Among the effects of gastroplasty (vertical ring and adjustable silicone gastric banding) after 6 months of follow-up, no major deficiencies have been observed, except low iron serum levels [15]. After one and two years of laparoscopic sleeve gastrectomy, patients with MO did not present iron deficiency comparing with baseline levels [16]. With the mini-gastric bypass, one of the most frequent long-term complications seems to be the iron deficiency anaemia (4.9%) [17]. One-anastomosis gastric bypass has been defined as a simple, safe and effective operation (with less perioperative risk than conventional gastric bypass) with only few cases of iron deficiency anaemia as long-term complication [18]. The gastric bypass applied to adolescents with MO seems to be an effective method for weight reduction with 50% of iron deficiency anaemia after a one-year follow-up [19]. In the case of Roux-en-Y gastric bypass, it has been observed that haemoglobin (from 13.4 g/dL to 12.8 g/dL) and ferritin (from 87.5 ng/mL to 55.4 ng/mL) decreased after surgery at 24–48 months whilst serum iron increased (from 68.4 μg/dL to 82.8 μg/dL) [20]. Another study based on the same technique reported low ferritin levels in 15% and anaemia in 17% one year after surgery [21]. With a longer period of follow-up, iron deficiency was observed in 40% and 54.5%, 2 and 3 years after surgery respectively. At 2-year follow-up, 46.6% of the patients had already developed anaemia and 63.6% at 3 years [22]. In a study based on the results of biliopancreatic diversion of Scopinaro and its modified technique, there was more prevalence of malnutrition and of iron deficiency in the Scopinaro group (16% and 60%) than in the modified group (2% and 40%) with a range of follow-up of 1–12 years [23]. Comparing different techniques (vertical banded gastroplasty, biliopancreatic diversion, modified biliopancreatic diversion and laparoscopic Roux-en-Y gastric bypass), iron deficiency was observed in 20%, 62%, 40% and 30.5% respectively after a follow-up between 4 and 12 years [24]. Considering the need of parenteral iron, it has been reported that patients in the biliopancreatic diversion/duodenal switch group had significant lower level of haemoglobin at presentation, relatively lower ferritin levels and required more additional parenteral iron treatment after the initial resolution of anaemia when compared with patients in the Roux-en-Y group. It must be noted that premenopausal women required earlier parenteral iron replacement [25]. In a retrospective study comparing patients with MO who underwent the Scopinaro technique or laparoscopic gastric bypass, it was observed that two years after the surgical procedure the most common nutritional complications were anaemia and iron deficiency, which occurred in 54.4% and 36.6% respectively [26].

### 3.2. Follow-up Studies after a Specific Surgical Technique

#### 3.2.1. Laparoscopic Sleeve Gastrectomy

In a study assessing the longer-term micronutrient status after this technique, it has been reported that patients had not relevant disturbances in iron parameters despite haemoglobin and haematocrit being less than normal in 28.6% and 25% after normalization from baseline by year 5. In this study 28.9% of patients were taken supplements in year 1, 42.9% in year 3 and 63.3% in year 5 [27]. Another study has shown that 48% of the patients were supplemented with iron. Seven of these patients developed microcytic anaemia. In this study, 25 out of 100 patients underwent a second bariatric intervention and in 23 of those who were supplemented the aim was as prophylaxis after the second technique [28]. With the same technique, Capoccia *et al*. [18] have reported the absence of iron deficiency and/or anaemia throughout 12 months and Pech *et al.* [29] used iron supplementation in 42 patients (51.2%) during a follow-up of 2 years. Six of them experienced microcytic anaemia. In this study no patient suffered from iron deficiency before operation and were with normal haemoglobin and iron levels preoperatively. In 23 of the 42 patients iron supplementation was performed as prophylaxis of a second intervention [29].

In a study including sleeve gastrectomy and gastric banding, there were not significant differences in serum ferritin and serum iron between baseline and after 6 months of the surgery. The haemoglobin and haematocrit status were improved. Again, in this study authors stated that patients acquired more of their dietary iron in the supplemental form after surgery [30].

Hakeam *et al.* [31] studied specifically the impact of laparoscopic sleeve gastrectomy on iron indices. In this case, after 1-year follow-up, the levels of iron and transferrin saturation were improved. Patients who participated in this study were given an iron-free multivitamin formula.

#### 3.2.2. Other Techniques

The prospective study by Kalfarentzos *et al.* [32] based on the vertical banded gastroplasty (average follow-up time 4.1 years) showed anaemia and iron deficiency in 46% and 32% of patients respectively despite the fact that they had been taking multivitamin and mineral supplements for at least 6 months. Comparing the results of the gastric bypass and control participants who underwent a lifestyle intervention, there were not significant differences with respect to iron after 1-year follow-up. The group of patients received multivitamin, iron, calcium, vitamin D, and vitamin B12 supplement [33]. With the laparoscopic gastric bypass, the preoperative and 1-year postoperative percentage of abnormal levels have been reported to be 16% and 6% for iron and 9% and 3% for ferritin respectively [34]. Considering the duodenal switch and with a median follow-up time of 30 months, several micronutrient deficiencies have been reported, the iron deficiency being 42.9%. In these cases, replacement therapy was necessary [35]. Vazquez-Prado *et al.* [36] have reported (postsurgical monitoring up to 60 months) iron deficiency in the third month after surgery (49.9%). This percentage decreased with the supplementation therapy, particularly after the second year post-surgery.

With respect to Roux-en-Y technique, iron deficiency has been reported to be 12.2% at the beginning of the study and 14.6% at the end (12 months) [37].

### 3.3. Follow-Up Studies Comparing Two Different Surgical Techniques

#### 3.3.1. Sleeve Gastrectomy *vs.* Roux-en-Y Gastric Bypass

In a recent study, heme-iron absorption was 23.9% before and 6.2% 12 months after surgery and nonheme-iron absorption decreased from 11.1% to 4.7%. No differences were observed by type of surgery. Iron intakes from all sources of supplements were 27.9 ± 6.2 mg/day in the sleeve gastrectomy group and 63.2 ± 21.1 mg/day in the Roux-en-Y group. Serum ferritin and total-body iron decreased more after Roux-en-Y technique than after sleeve gastrectomy [38]. Comparing laparoscopic sleeve gastrectomy and laparoscopic Roux-en-Y gastric bypass in a follow-up study between 3 and 36 months (mean follow-up time was 24.4), Gehrer *et al.* [39] reported fewer nutrient deficiencies after using the first technique than after using the last one. With regards to the iron deficiencies after surgery there were not significant differences comparing the two techniques (18% after sleeve gastrectomy and 28% after Roux-en-Y). Another study has included biliopancreatic diversion with duodenal switch besides sleeve gastrectomy and Roux-en-Y technique with a follow-up between 6 and 24 months. With respect to iron, the percentages of iron deficiency patients were 52% pre-surgery and 33.9%, 30.8% and 37.7%, 6, 12 and 24 months after surgery. The mean transferrin saturations (%) were 20.1, 23.5, 23.9 and 22.2 respectively. The percentages of iron supplementation were 2.2%, 55.4%, 60.7% and 65.2% respectively. Comparing the three techniques, the percentages of iron deficiency patients were 50.2% (pre-surgery), 29.9% (after 6 months) and 29.5% (after 12 months) for Roux-en-Y, 76% (pre-surgery), 71% (after 6 months) and 58.3% (after 12 months) for duodenal switch and 21.7% (pre-surgery), 32.1% (after 6 months) and 25% (after 12 months) for sleeve gastrectomy [40].

#### 3.3.2. Roux-en-Y Technique *vs.* Other Techniques

Comparing the Roux-en-Y technique with laparoscopic biliopancreatic diversion with duodenal switch, no differences were found with respect to iron deficiency after 1-year follow-up study. At the end of the study, 65% and 67% of patients were taken iron supplements in the case of gastric bypass and duodenal switch respectively [41]. Skroubis *et al.* [42] compared these two techniques and with respect to the percentages of iron deficiency they were 25% and 100% for Roux-en-Y and biliopancreatic diversion respectively after 5 years. The same percentages were obtained with regards to ferritin.

### 3.4. Other Comparisons

Coupaye *et al.* [43] analyzed the nutritional consequences of adjustable gastric banding and gastric bypass in 1-year follow-up study. In the case of the iron, there was a significant difference with 14% and 22% of patients having iron deficiency for gastric banding and gastric bypass respectively. The percentages of transferrin saturation lower than 20% were 52% and 37% respectively [43].

In other studies the results of a technique after having failed another one have been analyzed. Thus, the results of biliopancreatic diversion with duodenal switch after a previous laparoscopic gastric banding were analyzed in a 3-year follow-up study. There were deficiencies that either increased, remained stable or decreased, iron and ferritin deficiencies being some of those which remained unchanged [44]. Another similar study (biliopancreatic diversion after vertical banding gastroplasty) has shown that one out of twelve patient developed intractable iron-deficiency anaemia after a median follow-up of 21 months. 

### 3.5. Nutrition Care after Bariatric Surgery

It has been stated that laparoscopic sleeve gastrectomy may be preferred over Roux-en-Y because it is a less complicated operation. Nevertheless, patients should take daily micronutrient supplements (potentially including iron) to prevent deficiencies [45]. Iron deficiency has been considered as common with the three well-known types of technique (adjustable gastric bands, sleeve gastrectomy and Roux-en-Y gastric bypass), so a life-long follow-up of all patients besides virtually routine mineral and multivitamin supplementations are key points after BS [46]. With respect to iron deficiency and iron deficiency anaemia, the monitoring should continue indefinitely even after the initial iron repletion and anaemia resolution. In addition, parenteral iron treatment should be provided as required [7] especially in premenopausal women [25]. It must be noted that generally malabsorptive procedures can result in serious nutritional problems when patients do not take required supplements after BS, iron deficiency being one of the most common deficiencies [47]. BS in adolescents shows similar short- and long-term complications comparing with adults including iron deficiencies [48]. Especial attention should be given to adolescents, mainly girls at reproductive age who have a higher substantial risk of developing iron deficiency [49]. Despite being recommended, some authors have reported that iron supplementation after BS has been disappointing [50]. Repeatedly, iron deficiency has been on of the most common after BS besides others like folic acid, calcium, vitamin D, protein and vitamin B_12_. Correlating different degrees of preoperative malnutrition with postoperative nutritional complications may be very helpful in deciding which parameters to monitor [24,51]. Specific protocols are required to monitor vitamin and mineral deficiencies taking into account that the risk for postoperative undernutrition has been evidenced up to 1 year while spontaneous improvement in food intake has been observed as slow and inefficient [52]. It has been suggested that annual laboratory studies are required following any type of bariatric operation, this being sufficient to identify unfavorable trends [53]. Fortunately, despite wide variations in the performance of laboratory tests and the use of supplements, the practice patterns of most surgeons protect patients from developing severe deficiencies after procedures like Roux-en-Y gastric bypass or biliopancreatic diversion [54].

## 4. Discussion

Some authors have highlighted that few data exist concerning preoperative nutritional status in patients undergoing BS [55,56]. It has been reported that is not rare to find nutrient deficiencies prior to surgery [57]. Generally overweight and obesity are risk factors for the deficits of several micronutrients [58] and with respect to iron the prevalence of preoperative deficiency has been reported to be 35% [59]. Iron depletion previous to BS among obese patients has been related to high levels of inflammatory markers and BS seems to correct this iron depletion by means of a reduction of the chronic inflammation [60,61]. In fact there are several studies suggesting a potential link between obesity and altered iron metabolism [62]. 

Data after surgery seem to be inconsistent taking into account that the three main variables have not always been similarly controlled among studies. Thus, the patients’ preoperative status may be significantly different whilst the follow-up periods are different too. In addition, while some authors report differences with respect to iron deficiency and iron deficiency anaemia based on different techniques, others have not reported such as differences. With respect to the data, the methodological differences lead to figures of those deficiencies with a range between 5% and 65% depending on the studies. Another point to discuss is that preventively the majority of interventions after surgery include vitamin and mineral supplementations, thus being difficult to know the negative effect of operation. 

There is a consensus that a regular follow-up after BS is required [63,64,65] as well as preoperative deficiencies’ detection and correction [66]. The long-term goal is to avoid weight regain as well as to avoid deficiencies especially of protein, calcium, vitamin D, vitamin B12 and iron (D45). In this regard the role of the general practitioner has been emphasized [12,46,47,63].

There are several reasons to explain iron deficiencies after gastric bypass such as intolerance of red meat, diminished gastric acid secretion and exclusion of the duodenum from the alimentary tract. It seems that menstruating women, pregnant women and adolescents may be particularly predisposed toward developing iron deficiency and iron deficiency anaemia after bypass surgery [67]. The aim of the BS is making the patient loose weight and thus post-surgery diet is usually designed to achieve that goal. However, nutritional education after surgery must take into account not only the patient’s weight but also the possible nutrient deficiencies [68] especially in specific populations such as pregnant women [69] or adolescents [49].

The long-term follow-up after BS not only includes the periodical nutritional assessment but the nutrient and mineral supplementation when required [70,71,72,73]. Despite having stated that deficiencies appear to be more substantial following malabsorptive procedures [73], even less traumatic surgical approaches should not be considered free of risk [74]. A factor that needs to be taken into account refers to the adherence and compliance with the instructions after BS. In this regard, it has been shown that poor food choices increase after BS comparing with the pre-surgery elections (37% *vs.* 11%) [75]. Noncompliance with postsurgical nutritional regimens has been estimated to occur in from one third to almost two thirds of cases [76]. Considering the postoperative micronutrients deficits, recommendations for appropriate supplements and monitoring compliance are imperative [46]. It seems that post-surgery therapeutic patients education programs might be useful for increasing patients’ long-term compliance, which is often poor [46]. In the case of the iron, it must be noted that compliance with oral iron supplementation is usually no good, and once iron deficiency has developed it may prove refractory to oral treatment [7].

Different studies on micronutrients deficiencies after BS have reported not only iron deficiency but also others involved in iron metabolism. Thus, a significant reduction in serum vitamin C after 24 months after Roux-en-Y BS has been described [77]. Nevertheless other authors have not reported such a deficiency, so it remains controversial [33]. Anyhow, postoperatively oral iron prophylaxis and vitamin C in addition to a multivitamin should be prescribed for bypass patients, especially for vulnerable populations [67].

There are not standardized nutrition guidelines for use in BS but there are several recommendations with respect to patients’ follow-up and micronutrients supplementation [78,79,80]. The preoperative assessment should include a complete medical history, physical examination and laboratory testing. The routine screening of the presence of H. Pylori is not accepted unanimously. Finally, all patients should undergo a complete nutritional evaluation. With regards to the postoperative follow-up, there may be differences based on the surgical procedure but a concrete consultation schedule must be considered (every three months, every six months, *etc*.). Laboratory tests must include a complete evaluation for possible iron deficiency and the consequent anaemia. The lifelong daily micronutrients supplementation must be considered too with specific instructions for iron supplementation [7].

In summary, preoperative assessment of candidates for bariatric BS (medical and nutritional) as well as a well-established lifelong follow-up program is needed to detect and correct nutritional deficiencies and particularly iron deficiencies. 

## 5. Conclusions

Following BS, a relevant improvement in associated comorbidity is expected, especially with respect to diabetes, hypertension, dyslipidaemia and apnoea. Nevertheless, the postoperative complications are frequent as consequence of the alteration of the nutrients’ natural absorption [2,6].

Generally, a prevalence of anaemia and iron deficiency of between 10% and 40% has been estimated in candidates for BS. In the postoperative status the prevalence seems to be higher [8,9,10,11].

Comparing different surgical procedures, the prevalence of iron deficiency and anaemia varies depending on the follow-up time period and the specific characteristics of the population [24,25,26].

Patients’ preoperative status, differences among procedures, different micronutrients supplementation among others are variables, which contributes to yield very different data with respect to iron deficiency after BS.

It must be noted that a potential link between obesity and altered iron metabolism has been suggested [62].

There is a consensus that a regular follow-up after BS is required as well as preoperative deficiencies’ detection and correction [63,64,65,66].

Menstruating women, pregnant women and adolescents are particularly predisposed toward developing iron deficiency and anaemia after BS [67,68,69].

The lifelong supplementation of daily micronutrients must be considered, with specific instructions for iron supplementation.
